# Genotypic analysis of *Plasmodium falciparum* malaria parasites in a clinical trial of RTS,S vaccination in combination with seasonal malaria chemoprevention

**DOI:** 10.1186/s12936-026-06005-9

**Published:** 2026-07-02

**Authors:** Emily LaVerriere, Katrina Kelley, Alassane Dicko, Issaka Sagara, Almahamoudou Mahamar, Jean-Bosco Ouedraogo, Issaka Zongo, Halidou Tinto, Daniel Chandramohan, Brian Greenwood, Daniel E. Neafsey

**Affiliations:** 1 Department of Immunology and Infectious Diseases, Harvard T.H. Chan School of Public Health, Boston, MA USA; 2 Infectious Disease and Microbiome Program, Broad Institute of MIT and Harvard, Cambridge, MA USA; 3 The Malaria Research and Training Center, University of Science, Technology and Techniques of Bamako, Bamako, Mali; 4 Institut des Sciences et Techniques, Institut de Recherche en Sciences de la Santé, Bobo-Dioulasso, Burkina Faso; 5 Department of Disease Control, London School of Hygiene & Tropical Medicine, London, UK

## Abstract

Both vaccination and seasonal chemoprevention are effective interventions against malaria, but both morbidity and mortality due to malaria remain high, particularly in sub-Saharan Africa. A recent clinical trial showed that the combination of these interventions provided substantial protection against malaria over a five-year period. We performed amplicon-based genotyping of highly polymorphic parasite antigens within 1530 samples from this clinical trial. We evaluated the complexity of infection within these samples, finding that participants who received both interventions had less complex infections (fewer genetically distinct parasite strains) than participants who received either intervention given alone. We also evaluated the prevalence of parasites matching the vaccine construct CSP, at the level of haplotypes, epitopes, and individual amino acid changes. We found no significant differences in the prevalence of vaccine-matching alleles between vaccinated and unvaccinated groups. For context, this study genotyped about half as many non-vaccinated participants as the original phase 3 trial for RTS,S. While a comparable number of vaccinated individuals were genotyped in both studies, the infections in this study had less complex infections, leading to an overall lower number of parasite strains to analyze. These results suggest that the combination of vaccination with seasonal chemoprevention provides protection from infection, in addition to clinical disease, and they highlight the importance of continued molecular surveillance as malaria interventions are deployed.

**Supplementary Information:**

The online version contains supplementary material available at 10.1186/s12936-026-06005-9.

## Introduction

Malaria remains a persistent global health threat, causing an estimated 597,000 deaths per year, primarily in children under five years of age [1]. The burden of malaria is greatest in sub-Saharan Africa, where in many regions disease transmission is seasonal in nature due to rainfall-associated changes in the population size of *Anopheles* spp. mosquitoes that transmit the disease, as well as seasonal human behavioral changes [2]. While immunity to clinical disease has been demonstrated to accrue with age, acquisition of asymptomatic infections is age-independent, and occurs with similar timing across age groups following the commencement of the rainy season [3, 4].

Effective interventions for malaria in seasonal transmission settings are therefore timed to coincide with increased transmission, and include seasonal chemoprevention (SMC) and, more recently, seasonal vaccination. Seasonal chemoprevention usually involves multiple, regularly administered doses of sulfadoxine-pyrmethamine and amodiaquine during the expected transmission season [5]. Multiple vaccine candidates and schedules have been tested [6, 7], and the RTS,S/AS01_E_ and R21 vaccines are currently recommended for use by the WHO in malaria-endemic areas, each with a recommended three-dose primary schedule, followed by a booster dose [8, 9]. The timing of administration of the booster dose is usually age based but countries with highly seasonal malaria transmission may consider giving the booster dose prior to the malaria transmission season [9]. Both SMC and vaccination provide partial protection against disease that wanes over time [6, 10]. A recent clinical trial compared the combination of SMC and seasonal vaccination with the RTS,S/AS01_E_ vaccine (subsequently termed RTS,S) to each intervention on its own in areas with strongly seasonal malaria transmission [11, 12]. In this trial, the combination of SMC with RTS,S significantly reduced the incidence of uncomplicated disease, severe disease, and death compared to either intervention on its own.

In this study, we performed genotyping of highly polymorphic parasite antigens in participant samples obtained from clinical cases from children in each of the three arms of the combined SMC and vaccination clinical trial (vaccine only, chemoprevention only, and combination), in order to understand the nature of protection of the individual and combined interventions against infection. Our objectives were to determine whether the vaccine provided differential protection against parasites that had an exact match at the C-terminal of the circumsporozoite protein (CSP) antigen to the RTS,S vaccine construct, and to estimate the number of genomically distinct parasite lineages within each sample as a measure of protection against infection. Previous large-scale genotyping studies have examined the parasites present in infections from multiple RTS,S clinical trials [13, 14]. The initial genotyping analysis of the phase 3 trial found increased vaccine efficacy against parasite strains matching the CSP genotype used within the vaccine. More recent work analyzing a phase 2b clinical trial comparing different vaccine dosing regimens found that all vaccine regimens tested blocked some parasite infections, and that complexity of infection was reduced in participants in the RTS,S arm compared to the control arm.

In this study, we generated parasite genotyping data from samples meeting the clinical case definition for malaria from all three arms of the combined SMC and vaccination clinical trial [11, 12], allowing us to profile the number of clones in each. While the original study demonstrated a clear effect of the combined interventions on clinical disease, these newly generated genotyping data demonstrate evidence of combined efficacy against infection, without a signal of strain specificity.

## Methods

### Samples/original trial

We processed samples from the clinical trial NCT04319380, registered on ClinicalTrials.gov, as previously described [11, 12]. This study was a double-blind, individually-randomized, controlled, non-inferiority and superiority phase 3 trial done at two sites: the Bougouni district and neighboring areas in Mali and Houndé district, Burkina Faso. The original trial protocol was reviewed and approved by the ethics committees of the London School of Hygiene & Tropical Medicine, UK; the University of Science, Techniques, and Technologies, Bamako, Mali; the ethics committee for health research, Burkina Faso; and the regulatory authorities in Burkina Faso and Mali.

**Fig. 1:**
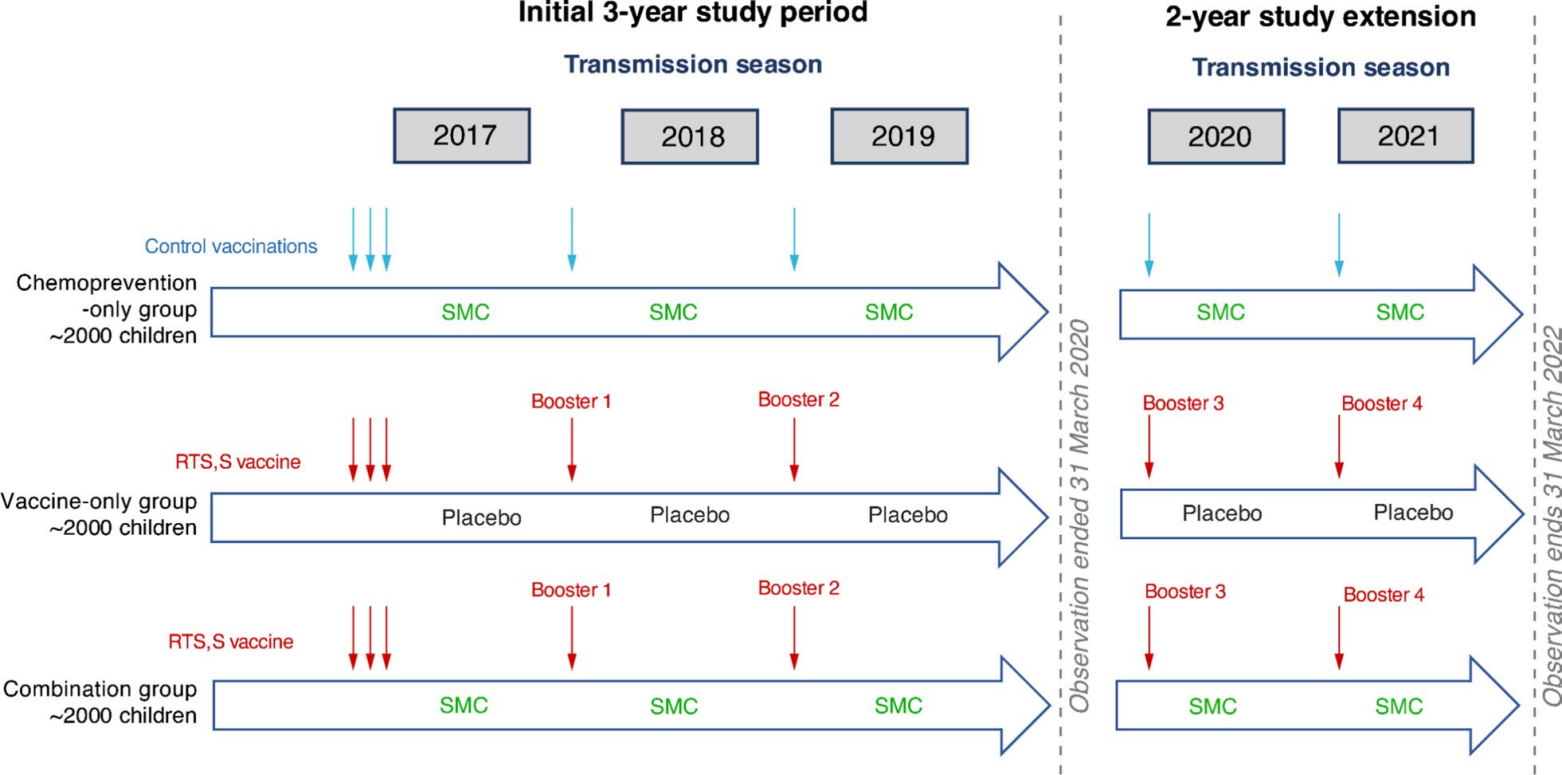
Study design of the original five-year trial. Adapted from Dicko & Ouedraogo et al. 2024 [12] under CC BY 4.0

Briefly, 6,861 children 5 to 17 months of age were enrolled and randomly assigned to receive either sulfadoxine-pyrimethamine and amodiaquine (the chemoprevention-alone group, 2,287 children), RTS,S/AS01_E_ (the vaccine-alone group, 2288 children), or both therapies (the combination group, 2,286 children). The chemoprevention-alone group received tetanus or tetanus-diphtheria toxoid vaccine in place of RTS,S/AS01_E_, and the vaccine-alone group also received sulfadoxine-pyrimethamine and amodiaquine placebos. Participants each received five doses of RTS,S/AS01_E_ or placebo vaccination: three in April-June 2017, followed by annual doses in 2018 and 2019. Participants each received four courses of sulfadoxine-pyrimethamine and amodiaquine or placebo per year. All participants received a long-lasting insecticide-treated bed net when enrolled into the study. An overview of the study design is in Fig. 1.

Uncomplicated malaria was the primary outcome of the original trial, defined as fever (either measured at least 37.5 ℃ or fever within the previous 48 h) and *P. falciparum* parasitemia of at least 5000 parasites per mm^3^. Participants presenting to trial health facilities with suspected malaria were tested with rapid diagnostic tests, and those with positive tests received artemether-lumefantrine and gave a blood sample for microscopic confirmation.

The initial trial lasted three years [11], and participants who did not have severe reactions to the interventions (febrile convulsions after vaccination) were invited to re-enroll in the extension study for the subsequent two years [12]. In 2020, 5098 children consented to re-enroll in the extension study; these participants retained the same treatment groups as before, resulting in 1,683, 1,705, and 1,710 participants in the extension study combination group, vaccine-only group, and chemoprevention-only groups, respectively. Participants no longer received SMC when they reached the age of five years, in line with national guidelines, and seasonal vaccination was no longer given after they reached this age. Thus, participants who enrolled when aged 5–11 months received two additional doses of RTS,S/AS01_E_ or placebo vaccination in June 2020 and June 2021, as well as four cycles of sulfadoxine-pyrimethamine and amodiaquine or placebo per year in 2020 and 2021, whilst those enrolled when aged 12–17 months received only one additional year of SMC and seasonal vaccination in 2020.

A power calculation (two sample test for proportions) for comparing the prevalence of the 3D7-matching haplotype between groups estimated that with a significance threshold of 0.05, estimated prevalence of 3D7-matching haplotypes of 0.15 in the non-vaccinated population, and a potential difference of 0.05 in prevalence, the study would have 80% power with 500 samples per group. To select samples for sequencing, samples which came from infections meeting the clinical definition of malaria in the original trial (symptomatic disease with ≥ 5,000 parasites/μL identified via microscopy) were stratified by intervention, country, and year. Years were limited to 2017, 2019, 2021, rather than including samples from all five years of the study, to avoid decreasing the statistical power of the stratified groups. 1 in 2 samples per stratification were randomly selected for inclusion, which resulted in 730 samples from the vaccine-only group, 572 samples from the chemoprevention-only group, and 228 samples from the combination group (Table 1). Sequencing was attempted on all selected samples, which were randomly assigned to different plates before beginning DNA extraction, to minimize the potential bias of any plate-based batch effects. Metadata on each sample on which sequencing was attempted is included in Supplemental Metadata.

**Table 1:** Number of samples per group, year, and country

Country	Burkina Faso			Mali		
Year	RTS,S	SMC	RTS,S + SMC	RTS,S	SMC	RTS,S + SMC
2017	114	50	12	85	76	10
2019	204	182	61	65	82	37
2021	154	71	58	108	111	50
Total	472	303	131	258	269	97

Number of samples per group, year, and country. All samples were from infections meeting the clinical definition of malaria in the original trial (symptomatic disease with ≥ 5000 parasites/μL identified via microscopy). “RTS,S” refers to the vaccine-only group, “SMC” refers to the chemoprevention-only group, and “RTS,S + SMC” refers to the combination group.

### Sequencing

We extracted genomic DNA from the dried blood spot samples and prepared them for sequencing using the 4CAST amplicon panel, as previously described [15], with no changes in protocol from the original publication. This amplicon panel targets four highly polymorphic regions of the *P. falciparum* genome, within the *csp, ama1, sera2*, and *trap* genes. Particularly relevant for this study, the amplicon within *csp* covers the polymorphic C-terminal region that is part of the RTS,S vaccine construct (nucleotides 1,487 through 1,819 within *csp;* Pf3D7_03_v3:221327–221659). The coverage of this region of *csp* allows for direct identification of parasites matching or mis-matching the CSP C-terminal of the vaccine strain in this region. While this amplicon does not cover the NANP repeat region of CSP that is also part of the RTS,S vaccine construct, the high diversity captured within this amplicon panel also allows estimation of complexity of infection (COI; number of genotypically distinct strains within an infection) more accurately than a single amplicon alone. As each of the amplicons is between 250 and 350 bp, 2 × 250 bp paired-end sequencing reads will fully cover each amplicon, allowing for each read-pair to be analyzed as a haplotype (or allele), rather than looking at each variant individually.

Samples were physically randomized before DNA extraction, to minimize the chances of batch effects biasing results of any one set of samples. We sequenced 1,346 samples (88% of all samples) with Illumina NovaSeq instruments, dividing the samples evenly between two lanes. We sequenced a second batch of samples (n = 184) on an Illumina MiSeq instrument. All sequencing runs were paired-end, 2 × 250 bp reads. Samples were processed in 96-well plates, with each plate containing extraction and PCR controls that were carried through sequencing and analysis. Data from these samples were submitted to the NCBI Sequencing Read Archive (http://www.ncbi.nih.gov/sra) under accession PRJNA1345094.

### Analysis

We processed the data through the malaria amplicon pipeline, available at https://github.com/broadinstitute/malaria-amplicon-pipeline [15] This pipeline pre-processes raw FASTQ files (adapter removal, primer removal, quality-trimming), which are then processed through DADA2, a denoising algorithm for amplicon sequencing data that compares the sequence variants observed to an expected error distribution and maps denoised reads to microhaplotypes (referred to as “haplotypes”). The pipeline then maps the denoised haplotypes to a custom database of expected haplotypes from the 3D7 reference sequence for each amplicon and summarizes the reads per haplotype per sample. We ran the pipeline with the default settings (preprocess = 1; remove_primer = 1; Class = parasite; dada2_default = 0; maxEE = “5,5”; trimRight = “0,0”; minLen = 30, truncQ = “5,5”; max_consist = 10; omegaA = 1e-120; matchIDs = 1; qvalue = 5; length = 20).

We set a threshold for the minimum number of reads per haplotype per sample at 200 reads (for samples sequenced on NovaSeq, which had a median of 184,000 reads and mean of 277,000 reads per sample) or 20 reads (for samples sequenced on MiSeq, which had a median of 25,000 reads and mean of 24,000 reads per sample). We also required a haplotype to have a within-sample frequency of at least 1% as estimated by sequencing reads, and we removed any haplotypes that appeared in only a single sample from the full dataset, to avoid PCR artifacts that occurred in early amplification cycles. Singleton filtering removed 463 of 11,557 haplotype detections (across all loci and samples), or about 4% of haplotypes detected.

We examined the positive and negative control wells present on every 96-well plate; positive control wells contained Dd2 genomic DNA, and negative control wells were present from DNA extraction through sequencing to detect contamination or potential index-hopping. We detected over 10,000 reads per expected Dd2 haplotypes per positive control well in all NovaSeq plates and over 1,000 reads per haplotype per well in all MiSeq plates. All haplotypes in the positive controls were exact matches to the expected Dd2 haplotypes (edit distance from Dd2 was zero). We did not detect any haplotypes present in negative control wells, except for in one plate which had thousands of reads of multiple haplotypes; all samples on that plate were removed from the final dataset. Besides that plate, only 22 samples did not generate data (either the reads per haplotype were lower than the threshold set, or the data were not of sufficient quality to call haplotypes.) These 22 samples did not appear to be biased towards any plate, well, study arm, or country of origin.

One of the two NovaSeq lanes had several sequencing artifacts. Specifically, three nucleotide changes within the CSP amplicon were detected in some samples:genomic coordinates 221,383, 221,363, and 221,354 on chromosome Pf3D7_03_v3 in the 3D7 reference genome (v3, PlasmoDB v46), all changed to C. Within *csp*, these coordinates are nucleotides 1763, 1783, and 1792, respectively, and they are within amino acids 378, 385, and 388. We inspected this region in previously generated amplicon sequencing datasets of parasites from similar regions, and none of these three changes appeared [3, 16]. We also looked within the Pf3k dataset of whole-genome data [17] and did not see these nucleotide changes in any samples. Finally, we selected eight samples where these nucleotide changes were present, re-amplified this region with the CSP primers, and confirmed with Sanger sequencing that the nucleotide changes were not present in the samples. We masked these three positions in all downstream analysis.

We performed all subsequent analyses on denoised haplotype calls, the output from the malaria amplicon pipeline as described above, in R 4.2.0, with the packages Biostrings, DescTools, easystats, grantham, here, RColorBrewer, stats, and tidyverse [18–25]. For each sample, we counted the number of distinct haplotypes called per 4CAST locus (CSP, AMA1, SERA2, TRAP). We estimated COI as the maximum number at any of these four loci per sample. While 77% (n = 1,170) of all samples had data at all 4CAST loci, this estimation of COI does not rely on having all loci present. All statistical tests were two-sided, unless otherwise noted. We tested for significance using the “kruskal.test”, “fisher.test”, and “p.adjust” functions from the stats package, as well as the “BinomDiffCI” function from the DescTools package.

## Results

### Clinical infections are less complex in individuals who received RTS,S/AS01_E_ with SMC

We generated high-quality *P. falciparum* sequencing data from 1530 samples in total, all of which came from infections meeting the clinical definition of malaria in the original trial: symptomatic disease with ≥ 5,000 parasites/μL identified via microscopy. Concordant with the original study [11], the number of clinical disease cases was much lower in the individuals who received both SMC and RTS,S than in either the vaccine-only group or the chemoprevention-only group, leading to an uneven number of samples to sequence between each study arm. Thus, we sequenced 228 samples from the combination group, compared to 730 samples from the vaccine-only group and 572 samples from the chemoprevention-only group (Table 1)

**Fig. 2:**
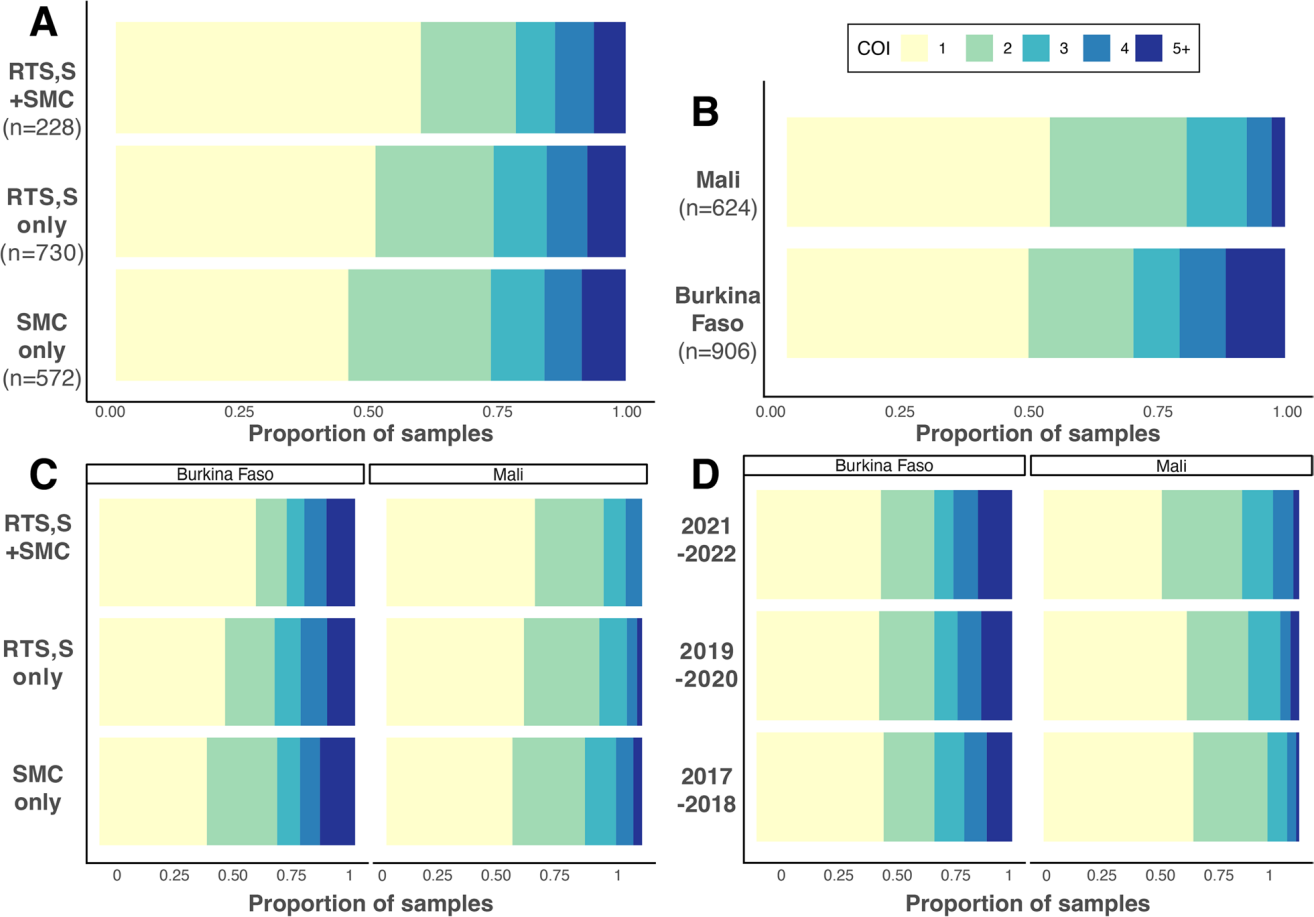
All panels visualize the proportion of samples at estimated complexity of infection (COI) level, from 1 (monoclonal) to 5 + (highly polyclonal). “RTS,S” refers to the vaccine-only group, “SMC” refers to the chemoprevention-only group, and “RTS,S + SMC” refers to the combination group. **A** Samples are stratified by study arm. Samples are more likely to have lower COI in the combination group (Kruskal–Wallis test, x^2^(2) = 9.11, P = 0.011, ε^2^ = 6.45^–3^ (95% CI [2.26^–3^, 1.00]); Dunn test with Holm correction, both vs. SMC only: adjusted P = 0.0079). **B** Samples stratified by country. COI tended to be higher in samples in Burkina Faso than in samples from Mali (Kruskal–Wallis test, x^2^(1) = 22.35, P = 1.41 e^−5^, ε^2^ = 0.01 (95% CI [6.35^–3^, 1.00]); mean COI in Burkina Faso = 2.3, mean COI in Mali = 1.8). **C** Samples stratified by study arm and country. Samples are significantly less complex in the combination group in Burkina Faso (Kruskal–Wallis test, x^2^(2) = 6.84, P = 0.03 ε^2^ = 8.75^–3^; Dunn test with Holm correction, both vs. SMC only: adjusted P = 0.027). Samples are less complex in the combination group in Mali, though not significantly (Kruskal–Wallis test, x^2^(2) = 3.76, P = 0.15). Samples per group in Burkina Faso: RTS,S + SMC, n = 131; RTS,S only, n = 472; SMC only, n = 303. Samples per group in Mali: RTS,S + SMC, n = 97; R,TSS only, n = 258; SMC only, n = 269. **D** Samples stratified by time period and country. COI distributions were consistent over time in Burkina Faso (Kruskal–Wallis test, x^2^(2) = 0.22, P = 0.90), but differed in later years in Mali (Kruskal–Wallis test, x^2^(2) = 8.85, P = 0.011; Dunn test with Holm correction, 2017–2018 vs. 2021–2022, adjusted P = 0.012). Samples per group in Burkina Faso: 2017–2018, n = 176; 2019–2020, n = 447; 2021–2022, n = 283. Samples per group in Mali: 2017–2018, n = 171; 2019–2020, n = 184; 2021–2022, n = 269

We first leveraged the genotyping data to identify complex (polyclonal) infections. Following filtering of haplotypes below 1% within-sample frequency, we estimated the complexity of infection, or the number of genetically distinct parasites identified in each sample. We found that monoclonal infections were more common in individuals who received both RTS,S and SMC than in those who received SMC only (Fig. 2A; Kruskal–Wallis test, x^2^(2) = 9.11, P = 0.011, ε^2^ = 6.45^–3^ (95% CI [2.26^–3^, 1.00]); Dunn test with Holm correction, combination group vs. chemoprevention-only group: adjusted P = 0.0079; combination group vs vaccine-only group: adjusted P = 0.082; vaccine-only group vs. chemoprevention-only group: adjusted P = 0.15.

We also compared COI distributions by country (Fig. 2B). Both countries had similar proportions of monoclonal infections—approximately half of all sequenced infections were monoclonal (48.5% in Burkina Faso (n = 393), 52.3% in Mali (n = 390). However, among the infections with more than one clone detected, COI tended to be higher in individuals in Burkina Faso than in individuals from Mali (Kruskal–Wallis test, x^2^(1) = 22.35, P = 1.41 e^−5^, ε^2^ = 0.01 (95% CI [6.35^–3^, 1.00]); mean COI in Burkina Faso = 2.3, mean COI in Mali = 1.8). When stratified by both study arm and country (Fig. 2C), COI distributions follow a similar trend as in Fig. 2A. In Burkina Faso, the pattern of monoclonal infections being more common in the combination group was observed again (Kruskal–Wallis test, x^2^(2) = 6.84, P = 0.03, ε^2^ = 8.75^–3^ (95% CI [2.42^–3^, 1.00]); Dunn test with Holm correction, combination group vs. chemoprevention-only group: adjusted P = 0.027). Monoclonal infections remained more common in individuals in the combination group in Mali as well, though the trend did not reach the threshold of statistical significance (Kruskal–Wallis test, x^2^(2) = 3.76, P = 0.15).

COI distributions remained consistent over time in Burkina Faso (Fig. 2D; Kruskal–Wallis test, x^2^(2) = 0.22, P = 0.90). In Mali, monoclonal infections became less frequent in later years (Kruskal–Wallis test, x^2^(2) = 8.85, P = 0.011, ε^2^ = 0.01 (95% CI [3.90^–3^, 1.00]); Dunn test with Holm correction, 2017–2018 vs. 2021–2022, adjusted P = 0.012). As previously reported, malaria transmission was consistently higher in Burkina Faso than in Mali, as measured via a cross-sectional survey of children not participating in the trial [11, 12]). The prevalence of *P. falciparum* at the end-of-season cross-sectional surveys in the original study was also lower in the chemoprevention-only group in 2017 than in subsequent years (1.7% prevalence in 2017, 15.5% prevalence in 2019, 8.2% prevalence in 2021) [11, 12].

### Parasites matching the vaccine strain appear at similar proportions in all groups

**Fig. 3:**
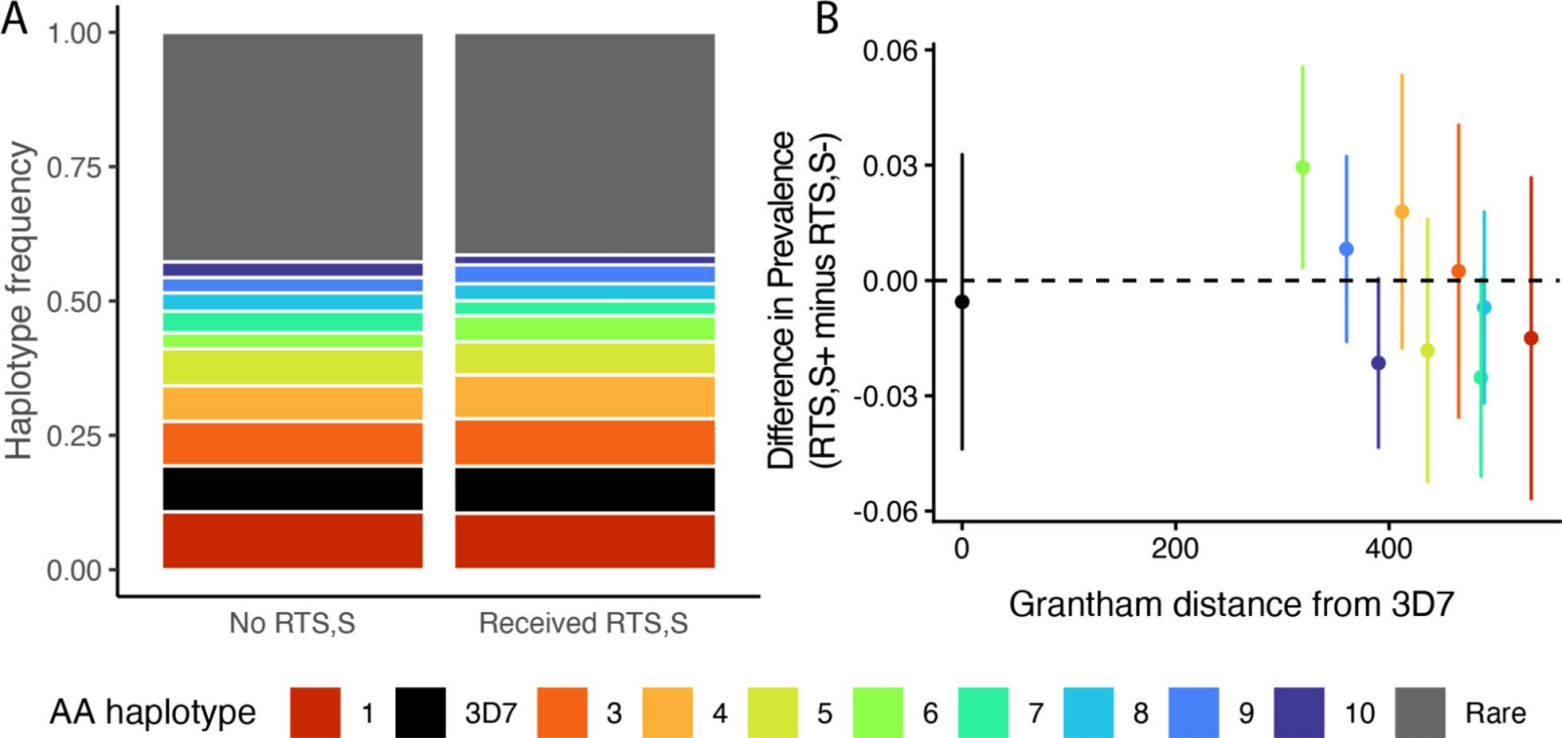
**A** Frequency of the top ten most common amino acid CSP haplotypes (each with a distinct color), including the 3D7-matching haplotype (second most common, colored in black). All less common haplotypes are grouped together and colored in grey. The corresponding amino acid haplotype sequences are listed in Supplemental Table S2. This region spans nucleotides 1,487 through 1,819 within *csp*; Pf3D7_03_v3:221327–221659. 3D7-matching haplotype prevalence did not differ between vaccinated and unvaccinated participants (Fisher’s exact test, one-sided, P = 0.386). **B** Grantham distance for each haplotype compared to the 3D7 haplotype is on the x-axis (distance is a sum of the Grantham distance from each non-3D7-matching amino acid per haplotype). The difference in prevalence of the haplotype between vaccinated and unvaccinated groups is on the y-axis. The lines spanning each point plot the 95% confidence interval around the difference in prevalences

**Fig. 4:**
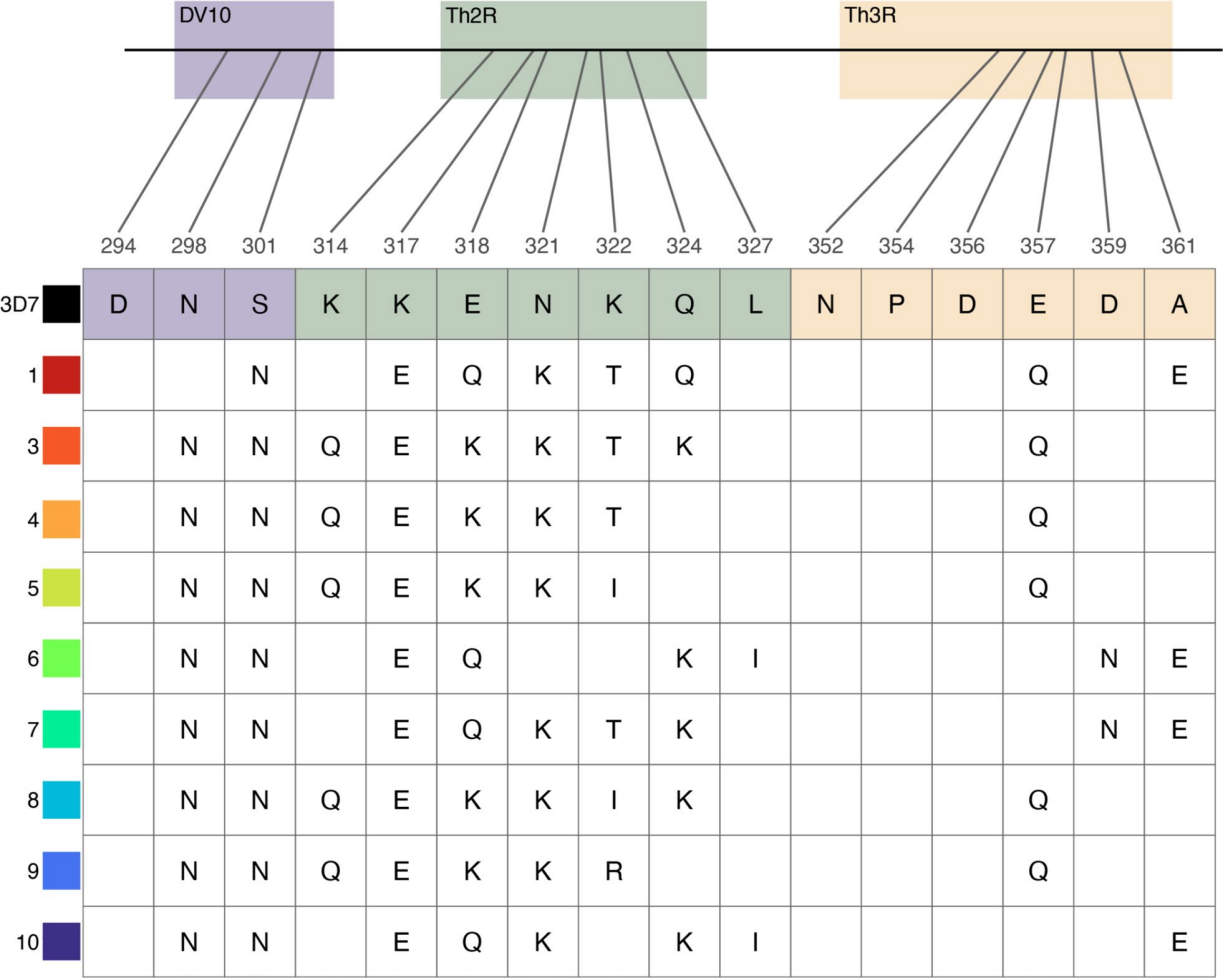
Most common CSP haplotypes observed. The line at the top denotes the region of CSP that was sequenced, with lines connecting to the table below where the observed variants fall. The three epitopes within this region are shaded in purple (DV10), green (Th2R), and yellow (Th3R). The first row of the table displays the amino acids at each position within the 3D7 reference sequence. Each of the following rows displays a commonly seen haplotype, labeled by its rank in the population (1–10; the 3D7-matching haplotype was the second most common) and a color matching those used for the same haplotypes in Fig. 3. In the non-3D7 rows of the table, only amino acids that do not match 3D7 are shown

After using all four loci in the amplicon panel to estimate complexity of infection, we focused solely on the haplotypes called from the CSP locus to examine the region of the vaccine construct that the amplicon covers. We compared the prevalence of CSP haplotypes matching the 3D7 vaccine strain between participants who received RTS,S and those who did not (Fig. 3a). Prevalence of amino acid haplotypes matching 3D7 did not differ between vaccinated participants (127 3D7-matching infections, prevalence = 14.4%) and unvaccinated participants (81 3D7-matching infections, prevalence = 15.1%; Fisher’s exact test, one-sided, P = 0.386). Additionally, we found no difference when considering the frequency of 3D7-matching haplotypes in place of prevalence; 3D7-matching haplotypes in vaccinated participants had a prevalence of 8.73% among all haplotypes, compared to a prevalence of 8.56% among non-vaccinated participants (Fisher’s exact test, one-sided, P = 0.579). Full lists of nucleotide and amino acid haplotypes observed in participants stratified by vaccination status are shown in Supplemental Tables 1 and 2. We also considered how similar the common haplotypes were to 3D7, by summing the Grantham distance of each mismatched amino-acid in a given haplotype (Fig. 3b). None of the most common haplotypes were very similar to 3D7 (sum of Grantham distances ranged from 319 to 533 per haplotype). Figure 4 shows the variant amino acids in each of these haplotypes.

We also evaluated whether evidence of allele-specific protection from clinical breakthrough infections could be identified at the three epitopes previously described for CSP: DV10, Th2R, and Th3R, rather than at the full haplotype level (Table 2). We compared the prevalence of amino acid haplotypes spanning each of these epitope regions individually, and we again found no difference associated with vaccination status forTh2R, Th3R, or DV10 (Fisher’s exact tests, one-sided; P = 0.641, 0.609, 0.778, respectively). The incidence of each epitope haplotype per study arm is described in Supplemental Tables 3–5.

**Table 2:** Prevalence of 3D7-matching CSP alleles in vaccinated (RTSS +) and unvaccinated (RTSS -) populations

Locus	3D7-matching allele prevalence			P-value
	RTSS +	RTSS-	Difference	
CSP	0.147	0.152	− 0.005	0.386
Th2R	0.157	0.162	− 0.005	0.641
Th3R	0.214	0.223	− 0.009	0.609
DV10	0.177	0.173	0.004	0.778

Prevalence of 3D7-matching CSP alleles in vaccinated (RTSS +) and unvaccinated (RTSS-) populations. The Locus column describes the allele being compared, including the full amino acid haplotype (CSP) and the amino acid haplotypes for each of the three CSP epitopes (Th2R, Th3R, and DV10). P-values are from Fisher’s exact test, one-sided.

Finally, we investigated individual amino acid positions within these epitopes to look for evidence of 3D7-specific protection. Specifically, we examined amino acid positions 294, 298, 301, 314, 317, 318, 321, 322, 324, 327, 354, 356, 359, and 361, which all vary in these populations, consistent with previous deep sequencing studies of *csp* [14, 16]. We examined each position on its own, and we compared the distributions of amino acids observed at that position across each of the study arms. The prevalence of the 3D7-matching allele at position 321, within the Th2R epitope, was higher in vaccinated participants (n = 281, prevalence = 0.282) compared to unvaccinated participants (n = 151, prevalence = 0.244. While the initial hypothesis testing was significant (Fisher’s exact test, one-sided, P = 0.01), after adjusting for multiple hypothesis testing, the result was not statistically significant (adjusted P = 0.22, after Benjamini–Hochberg correction). None of the other distributions varied significantly from each other, even before correcting for multiple hypothesis testing (Fisher’s exact test applied to each residue, prevalences and p-values in Supplemental Table 6, raw number of appearances of each residue in Supplemental Table 7).

## Discussion

In this study, we generated amplicon sequencing data to explore parasite diversity within symptomatic infections in a clinical trial which compared the combination of SMC with seasonal vaccination with RTS,S with either intervention given alone [11]. Genotyping data has the capacity to both confirm and, in this case, recapitulate the efficacy of the interventions in the trial. While the original study found an increase in protection against symptomatic disease in those who received both SMC and vaccination [11, 12], we found an additional benefit of the combined interventions. Participants who received both interventions and developed clinical disease were also more likely to exhibit lower complexity infections than those receiving only SMC or RTS,S alone (Fig. 2). This suggests that among participants who developed clinical disease, participants who received both interventions were more successful at preventing detectable infections, assuming comparable force of infection among study arms. This finding suggests that the combined chemoprevention and vaccination not only protects against clinical disease, but also has additive protection against symptomatic breakthrough infection itself, which can occur regardless of clinical symptoms. This change in parasite diversity among clinical breakthrough infections may manifest as lower complexity infections, as previously seen in individuals vaccinated with RTS,S [13].

The genotyping data generated also allowed us to look for differences in the strains within clinical infections as a result of vaccination, which was observed in the original phase 3 trial of the RTS,S/AS01_E_ vaccine [14]. In this dataset, we did not observe vaccination-related differences in the prevalence of 3D7-matching alleles when considering full haplotypes or epitopes. Several factors may have resulted in a greater temporal lag and a smaller impact of vaccination on the observed breakthrough infections genotyped here compared to the phase 3 trial. First, the administration of SMC in the combination group may have delayed the time to first infection within each season of transmission, thus leading to first infections occurring when vaccine efficacy has started to decrease. Second, the samples genotyped in this study were a random selection of all symptomatic cases (stratified by country and year), compared to the phase 3 trial which genotyped the first post-vaccination infection per individual. Finally, as this study was limited to clinical cases, any potential impact of vaccination on lower-parsitemia or asymptomatic cases can not be determined.

We did observe increased prevalence of the 3D7-matching CSP amino acid 321 N in vaccinated individuals, although this difference did not reach statistical significance after correcting for multiple hypothesis testing. However, we cannot rule out that a smaller magnitude difference in protection could be present below our limit of detection. A power calculation for a one-sided, two-sample test for proportion with 500 samples per group (1000 samples total),, estimated prevalence of the 3D7-matching haplotype of 0.15 in the unvaccinated population, and a significance threshold of 0.05, resulted in 80% power to detect a difference of ~ 0.05 in prevalence between groups. Any smaller differences in prevalence would require higher sample size. As vaccination rates increase throughout malaria-endemic regions, continued surveillance of malaria infections in vaccinated and unvaccinated populations will be necessary for detecting any signals of vaccine escape, and larger studies will be better powered to estimate the magnitude of any allele-specific protective efficacy that is present.

## Conclusion

Combining seasonal malaria chemoprevention with vaccination protected against clinical disease in a previous clinical trial. In the study reported in this paper, we found that the combination of SMC and seasonal vaccination also led to reduced levels of polyclonality within samples from participants with clinical disease, indicating increased protection against the establishment of high-parasitemia infection itself relative to participants receiving only one intervention. Within clinical breakthrough infections, infection polyclonality was similar between the vaccine-only and chemoprevention-only groups, suggesting comparable protection against infection provided by each intervention given alone, as was noted in analysis of clinical infections. These genotyping results underscore the additive value of vaccination and SMC for protecting children against malaria in seasonal transmission settings. In the vaccine-only and combination groups we were unable to detect significantly greater protection against parasites with *csp* C-terminus sequences matching this portion of the 3D7 vaccine strain, suggesting that such protection is modest in magnitude if present. Nevertheless, we advocate continued surveillance for evidence of vaccine escape as RTS,S and R21 are more widely deployed in Africa, when power to detect allele-specific protection may be enhanced.

## Supplementary Information

Supplementary Material 1.

Supplementary Material 2.

## Data Availability

Raw sequencing data generated in this study have been deposited in the NCBI Sequencing Read Archive with the accession PRJNA1345094.
